# The National Medical Union (Official)

**Published:** 1914-04-25

**Authors:** 


### THE NATIONAL MEDICAL UNION (Official).

Offices : 346 Strand. Tel.: City 8444.

### DR. MACEVOY ON THE CONSTITUTION.

The following notes on constitution have been prepared by Dr. Macevoy, of Brondesbury, and are published for the information of members on a subject which it is one of the duties of the Provisional Council to deal with.

---

We may assume that the National Medical Union is to be a democracy with universal suffrage, as this seems to be an indispensable condition of its success. The next step must be the formation of separate local bodies corresponding to the divisions of the British Medical Association.

The criticisms directed against the work of the divisions of the British Medical Association, against the divisions themselves, against the British Medical Association itself, have often been, to my mind, unfair. They should have been directed against the members—that is to say, the doctors. Divisions of the British Medical Association are often badly attended: there is frequently an attendance of only fifteen, twenty, or twenty-five per cent. of the membership. A large proportion of the most successful and busiest members do not take any great part in the management of affairs: control is usually in the hands of a handful of clever and energetic members. All these objections arise from the apathy of medical men, and from the abstention of members who do no work for the general good, either because they take no interest in the affairs of their profession or because their professional work takes up too much time.

In the formation of separate local bodies (constituencies, divisions) the National Medical Union will have greater difficulties to face than the British Medical Association. Since there are fewer members there will be greater difficulty in determining boundaries so as to facilitate intercourse between, and meetings of, the members, and accessibility of places of meetings. If the constituencies are free to govern themselves as they shall think fit, the views of the constituencies can only be properly obtained at meetings where there is free discussion, where both sides of questions can be heard and judged, and voting takes place. Post-card votes are not often taken in the British Medical Association divisions, but they are an advantage on some occasions. This is an important point and requires careful consideration; it is, of course, a very debatable one.

The question of the post-card vote and that of a general referendum are closely allied. The vexatious delay and the expense of a referendum in the case of the British Medical Association, no doubt, explain why for all practical purposes it is, and has been, useless. If the expense were negligible, and if delay could be avoided, it would now and then be useful. Obviously, it should be rarely required.

If the areas and the boundaries of constituencies are carefully considered, I do not see the necessity of units such as the branches of the British Medical Association. I have been a member of the Branch Council (Metropolitan Counties) for years, and while in theory it seems desirable to have a Branch Council with power to co-ordinate the work of the divisions, in practice my experience has been that an enormous amount of useless work is done there, wasting much time with no practical result. The boundaries of constituencies should be so drawn up that the Committees of these constituencies should be able to negotiate with municipal bodies, with local authorities (Poor-Law, &c.) of various kinds. Where the co-operation of constituencies for some special object is necessary, joint committees can be formed; delegates can be sent from one constituency to an adjoining constituency to attend meetings; and constituencies can keep in touch with one another without the introduction of such a complication as the Branch Council.

The British Medical Association Branch Council has practically no control over its divisions, except financial, as the divisions are autonomous. It is perfectly easy for financial relations to be established directly between the constituencies and the central governing body (*e.g.* B. M. A. Council). If the postal referendum is accepted as part of the constitution of the National Medical Union it is easy to apply it to the whole or to a part of the Union, if for some special object two or more constituencies find it necessary to act together.

Assuming that autonomous constituencies must exist, the only way, it seems to me, to control and direct the policy of the Union and manage its affairs is by the formation of a representative body composed of members elected by the constituencies, and a council which shall be the executive. Undoubtedly the B. M. A. representative body is too large, but if constituencies were larger, and therefore their number smaller, the number of representatives would be less. At the same time there are great difficulties in reducing the size of the B. M. A. representative body without doing injustice to some constituencies, and there must be a limit to the size of a constituency if facilities are to be given to members to attend meetings. I believe the National Medical Union will find this question one of the most difficult to solve.

The representative body should be an assembly with full deliberating powers. The men selected by the constituencies should obviously be men of honour, who can be trusted to do their duty and carry out, as far as they know them, the wishes of their constituents. At the same time, in emergencies they must also be trusted to act for the best according to their judgment of the merits of questions before them. They must be empowered to delegate specific powers to their council or executive committee to take action upon their behalf when the representative body is not in session.

The logical way of electing the executive committee would seem to be by the representative body. This question raises that thorny point, "government by a single chamber." The National Medical Union will have to decide between the two modes of central government:—

(*a*) Having a representative body which shall elect its executive committee, or (*b*) directly appointing a strong central council to carry out what it believes are the views of the constituencies of the Union.

The B. M. A. Council, elected partly by the representative body, partly by the constituencies, has, of course, been freely criticised for its actions in the fight over the National Insurance Act, but its faults (lack of decision, errors of judgment, etc.) were not, I think, due to the constitution of the council, nor to its mode of election, but to its personal failings, to the weakness of the material and the faulty selection of the constituencies.

### THE HAMPSTEAD NON-PANEL ASSOCIATION AND THE REGISTRAR.

In connection with the correspondence between the Registrar of the General Medical Council and the Hampstead Non-Panel Association, published last week, the following letters have now to be added:—

GENERAL MEDICAL COUNCIL.

April 6, 1914.

The Hon. Secretary,
National Medical Union.

Dear Sir,—In reply to your letter of the 2nd inst., I have to say that I have taken note of the intention of the Union to publish the correspondence which has passed between myself as Registrar and the Hampstead Non-Panel Association, the object being "to indicate the attitude of the General Medical Council towards the general questions involved." The duty of the Registrar is to inform complainants of the procedure prescribed by the Council, under its Standing Orders, for the formulation of charges of "infamous conduct in a professional respect" brought against individual medical practitioners on the Register. My letters were written in fulfilment of this duty alone. They do not purport "to indicate the attitude of the General Medical Council towards the general questions involved." That attitude can be indicated only by the Council itself, by means either of the "Warning Notices" which it issues from time to time, or by means of the judgment it pronounces in the case of any individual practitioner, against whom a properly formulated charge of "infamous conduct" has been duly brought, and proved to the satisfaction of the Council. I would therefore ask that, if the correspondence is published, you will be so good as to include this letter.—Yours faithfully,

Norman C. King, Registrar.

NATIONAL MEDICAL UNION.

April 8, 1914.

The Registrar,
General Medical Council.

Dear Sir,—In reply to your letter of April 6, as the object in view in publishing this correspondence is not a personal one as between yourself and the Hampstead Non-Panel Association, no particular purpose would be served in publishing your letter of April 6, which I take as a personal explanation. In your letter of March 10 you state that you are acting under direction of the authorities of the Council. The ordinary interpretation of the correspondence therefore is that it indicates the attitude of the authorities of the Council.

Perhaps you will kindly refer to your letter of March 10, from which it is clearly to be inferred that you have laid the matter before the authorities of the Council and have been directed to make the reply therein contained.—Yours faithfully,

Robert Carswell, Hon. Secretary.

GENERAL MEDICAL COUNCIL.

April 9, 1914.

The Secretary,
National Medical Union.

Dear Sir,—In reply to your letter of the 8th instant, I have to say that our correspondence is not complete unless my letter of the 6th instant is included. In publishing the correspondence, if my last letter is suppressed by you, it will be my duty to send a copy for publication also.—Yours faithfully,

Norman C. King, Registrar.

Action Taken by the General Council of Medical Education.

[After the above letters were in type we received a letter from Mr. Norman C. King, registrar of the General Council of Medical Education and Registration, dated 21st instant, referring to the correspondence published in our issue of 18th instant, and enclosing a copy of his letter to the National Medical Union dated 6th instant as given above. Mr. Norman King explains that on the 9th instant he wrote to the National Medical Union to say that the correspondence in our former issue was not complete without his letter of the 6th instant, and that in publishing the correspondence, if that letter was suppressed, it would be his duty to send a copy for publication.—Ed. The Hospital.]

**Sir John Collie on State Medical Service.**

At a meeting arranged to promulgate the advantages of a State Medical Service, held recently at the Essex Hall, Strand, Sir John Collie proposed the main resolution: "That, in the opinion of the meeting, the panel system had failed, and a State Medical Service freely available to all was the only means by which the health of the nation could be maintained." Sir John Collie attributed the financial strain on the Insurance Act to wholesale malingering. He said that when the Act was extended, as was inevitable, to forty-four millions of people a National Medical Service must follow.

Professor Leonard Hill said that the whole profession should be engaged in the study of preventive medicine. Dr. Alfred Salter, of Bermondsey, said that he was a member of a firm of five partners, with an average of 1,400 per doctor. During the last year, they saw 89 per cent. of these patients. The average duration of consultation was about $3\frac{1}{4}$ minutes, of which $1\frac{3}{4}$ minute was taken up in writing.

